# Safe Sex Messages Within Dating and Entertainment Smartphone Apps: A Review

**DOI:** 10.2196/mhealth.5760

**Published:** 2016-11-08

**Authors:** Evelyn Tzu-Yen Huang, Henrietta Williams, Jane S Hocking, Megan SC Lim

**Affiliations:** ^1^ Melbourne School of Population and Global Health University of Melbourne Melbourne Australia; ^2^ Austin Hospital Austin Health Heidelberg Australia; ^3^ Melbourne Sexual Health Centre Alfred Health Carlton Australia; ^4^ Centre for Population Health Burnet Institute Melbourne Australia; ^5^ Department of Epidemiology and Preventive Medicine Monash University Melbourne Australia

**Keywords:** mobile apps, sexual health, STDs, sexually transmitted diseases, mobile health, mHealth

## Abstract

**Background:**

Smartphone apps provide a new platform for entertainment, information distribution, and health promotion activities, as well as for dating and casual sexual encounters. Previous research has shown high acceptability of sexual health interventions via smartphone apps; however, sexual health promotion apps were infrequently downloaded and underused. Integrating sexual health promotion into established apps might be a more effective method.

**Objective:**

The objective of our study was to critically review popular sex-related apps and dating apps, in order to ascertain whether they contain any sexual health content.

**Methods:**

Part 1: In January 2015, we used the term “sexual” to search for free apps in the Apple iTunes store and Android Google Play store, and categorized the sexual health content of the 137 apps identified. Part 2: We used the term “dating” to search for free geosocial-networking apps in the Apple iTunes and Android Google Play stores. The apps were downloaded to test functionality and to determine whether they included sexual health content.

**Results:**

Part 1: Of the 137 apps identified, 15 (11.0%) had sexual health content and 15 (11.0%) contained messages about sexual assault or violence. The majority of the apps did not contain any sexual health content. Part 2: We reviewed 60 dating apps: 44 (73%) targeting heterosexual users, 9 (15%) targeting men who have sex with men (MSM), 3 (5%) targeting lesbian women, and 4 (7%) for group dating. Only 9 dating apps contained sexual health content, of which 7 targeted MSM.

**Conclusions:**

The majority of sex-related apps and dating apps contained no sexual health content that could educate users about and remind them of their sexual risks. Sexual health practitioners and public health departments will need to work with app developers to promote sexual health within existing popular apps. For those apps that already contain sexual health messages, further study to investigate the effectiveness of the content is needed.

## Introduction

In recent years, the number of smartphone users has surged across the world and downloads of smartphone apps have grown significantly [1]. Nielsen’s monthly survey found that 71% of American mobile phone users owned a smartphone by mid-2014 [2]. Smartphone apps provide a new platform for information distribution and networking. By 2013, there were over 50 billion app downloads from the Google Play store, and more than 60 billion from the Apple iTunes store [3,4]. This platform creates various opportunities for health promotion activities such as distributing health-related information, offering resources for health care, and providing forums for sharing experiences [1,5,6]. The benefits of using apps for health promotion are many, including low cost to develop and operate, potentially widespread distribution, and convenience for both health care providers and health care seekers [1].

The availability of geosocial-networking smartphone apps—apps that use the global positioning system to locate their subscribers—has created a novel way of networking that is quick, cheap, and convenient [7-10]. Users can easily identify other users by physical proximity. While these geosocial-networking apps can be used for forming friendships and building a community, they are frequently used for dating and to facilitate the process of finding sexual partners [7,8,11,12]. By filtering user profiles, such as age, appearance, and interests, subscribers can select the type of partners they seek [13]. Grindr, a popular dating app that targets men who have sex with men (MSM), had more than 7 million subscribers globally by 2013, and the number is increasing [14]. Previous studies have found that users of these dating apps report more sexual contacts and more casual sexual partners [7,8,10-12,15]. Users also reveal significant increases in casual sex since starting online dating [16]. Rice et al reported that 75% of Grindr subscribers had had sex with people they met through the app, and 15% reported unprotected anal sex with sexual partners from Grindr [8]. The likelihood of young MSM engaging in unprotected anal intercourse was 3 times higher among Grindr users than nonusers [11], and users reported a higher prevalence than nonusers of ever being diagnosed with sexually transmissible infections (STIs) [9].

Sexual health interventions that are integrated with modern technologies have been successful [17-22]. Text messaging has been widely used to promote sexual health, including appointment reminders, partner tracing, and result notification [22]. Studies of Internet-based human immunodeficiency virus (HIV) infection interventions targeting MSM using online questionnaires and tutorial sessions revealed a reduced rate of unprotected anal intercourse and increased condom use [20,21]. In recent years, due to the increasing use of smartphones, apps designed to provide sexual health information and education are readily available on the market [23]. However, these apps are infrequently downloaded, have low user ratings, and are unlikely to reach the target groups [23].

Rather than creating new sexual health apps, leveraging established and popular apps may improve the distribution of health promotion information to a larger number of users [15]. Most important, integrating sexual health information within these apps can be an effective way to reach key populations, such as MSM or people who have casual sexual partners [15,24]. In addition, it is possible for health professionals to harvest global positioning system data from the apps and provide services according to users’ physical locations, such as referral to STI testing centers [15]. Several studies have suggested that young adults consider this approach acceptable [10,15,24].

The aim of this study was to systematically and critically review free sex-related apps (including all apps that have sexual content, such as sexual entertainment, sexual health information, and sex enhancement) and popular free dating apps, determine whether they contain any sexual health content, and, if so, what kind of information they provide to educate users about sexual health.

## Methods

This review was conducted in 2 parts: a review of sex-related apps and a review of dating apps. Ethical approval was not required, as the research did not involve participants.

### Part 1: Review of Sex-Related Apps

#### Search and Inclusion/Exclusion Criteria

The first part of the study was a content analysis of free sex-related apps. We used the term “sexual” to search the Android Google Play marketplace (Google Inc, Mountain View, CA, USA) and Apple iTunes store (Apple Inc, Cupertino, CA, USA) in January 2015. We conducted the search under the stores’ default algorithms, except that we filtered to search for free apps in the Android Google Play marketplace (as the choice was available). The search yielded 250 apps from Android Google Play marketplace and 263 apps from the Apple iTunes store. We then excluded all the paid apps from the Apple iTunes store. Apps were also excluded if they did not have an English-language interface, if they served the function of online dating, or if they were not related to sex (eg, the search found a “find your phone” app).

#### Data Extraction and Review Methods

We recorded the following information from the individual apps during the review: the app store category (eg, health and fitness, games, education), the app developer, and the user rating (the average of individual user ratings of 1 to 5). Apps were classified according to their primary purposes: “sex aid or sexual exploration” covered apps that provide ideas about sexual positioning or foreplay; “entertainment” included game apps or apps that calculate sexual compatibility based on horoscope; “sex education/information” encompassed apps designed to provide sexual health knowledge; “sexual assault/violence” included apps with the primary purpose of tackling sexual assault or violence or helping the victims of sexual violence; and “other” covered apps that did not fit into the above categories, including period tracking apps and apps for sex offender registries. Chi-square or Fisher exact tests compared the main purposes of the apps and presence of sexual health content between iTunes and Google Play apps. Apps were downloaded and reviewed in February or March 2015 by a single reviewer (ETH). All the functions of each app were tested and the outcome was categorized as having “sexual health content,” “sexual assault/violence information,” or “none” (Textbox 1).

### Part 2: Review of Dating Apps

#### Search and Inclusion/Exclusion Criteria

The second part of the study was a review of popular dating apps. We used the term “dating” to search the Apple iTunes store and the Android Google Play marketplace in January 2015. The first 50 free dating apps from each store were included. Apps requiring in-app purchase for basic functions such as receiving messages and online chats were excluded. We included an extra 3 lesbian dating apps that were available in both stores and had the most downloads according to the download numbers available in the Android Google Play marketplace.

#### Data Extraction and Review Methods

We downloaded and reviewed the apps by creating a user profile and testing the apps’ functions during April and May 2015. We used 1 iPhone (Apple Inc) and 1 Android phone (HTC; HTC Corporation, Taoyuan, Taiwan) to test the functions of each app. A female profile was created for each heterosexual app and lesbian app, and a male profile was created for MSM apps. We classified apps as containing no sexual health content and no safe dating tips if we found no relevant information after testing all the functions of the app, and logging in and out on 5 separate days. The following information was extracted from the apps: app store category (social, social networking, and lifestyle), user rating, and the name of the app’s developers. Apps were categorized into 4 groups based on their primary target groups as heterosexual, MSM, lesbian, and other (apps for seeking threesomes or group dates). Chi-square or Fisher exact test compared the presence of sexual health content between apps with different target groups.

**Definition of categories of sex-related apps.**

- Sexual health content
  - Information about sexually transmissible infections (STIs)
  - STI testing information or resources
  - Information about condom use or assistance locating condoms
  - Information about contraception
- Sexual assault/violence content
  - Identification of signs of sexual assault
  - Prevention of sexual assault
  - Medical and psychological care after sexual assault
  - Information about sexual assault
- None
  Containing none of the information listed above

## Results

### Part 1: Sex-Related Apps

**Figure 1:**
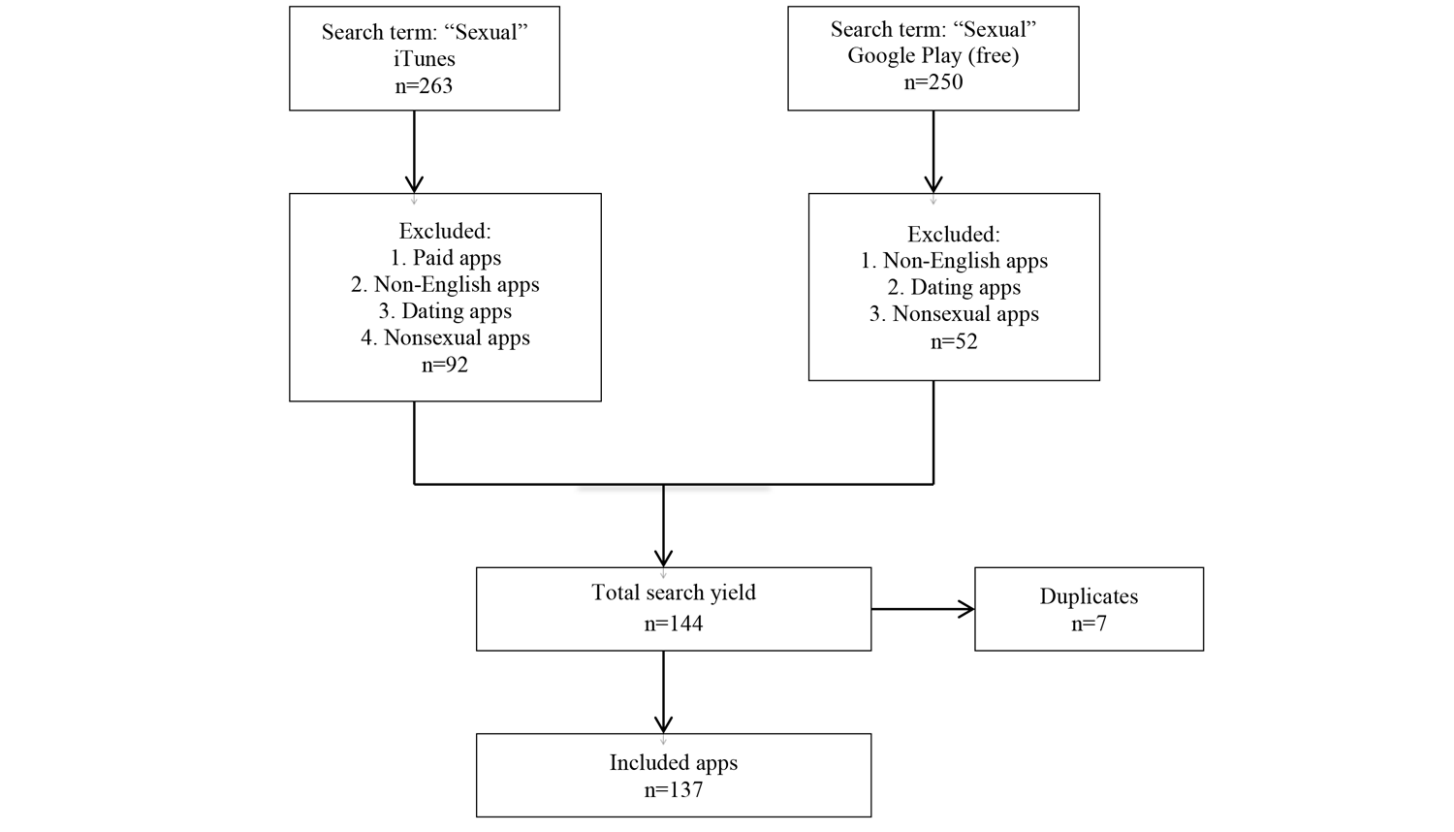
Inclusion of sex-related apps.

Our search yielded 250 apps from the Android Google Play marketplace and 263 apps from the Apple iTunes store. Ultimately, 137 apps were shortlisted for review (Figure 1). Of the 137 apps reviewed, the most common app purpose was sex aid and sexual exploration apps, which included information or advice on sexual positions (such as Kama Sutra apps) and apps that provided tips and ideas for foreplay and other techniques for promoting sexual pleasure (n=42, 30.7%). Other common categories were entertainment apps (n=32, 23.4%), apps relating to sexual assault (n=19, 13.9%), and apps for sexual education and information (n=12, 8.8%) (Table 1). A total of 15 apps (11.0%) included any sexual health content, and 15 apps (11.0%) contained sexual assault or violence content. iTunes apps were more likely than Google Play apps to have apps for the purpose of sex aids and sexual exploration or sexual assault (*P*=.01). Most of the apps (n=107, 78.1%) did not contain any sexual health content; that is, information about STIs, STI testing, condom use or assistance locating condoms, or contraception. There was no statistically significant difference in sexual health content between iTunes and Google Play apps (*P*=.06). Among the 15 apps that contained sexual health content, 5 (33%) had both contraception and STI information, 4 (27%) contained contraception information, and 6 (40%) contained information about STI and condom use for STI prevention. Most of the apps containing sexual health content were from the sex education and information category (n=11, 73%). The remaining sexual health information-containing apps were distributed as follows: 2 sex aid/sexual exploration apps, 1 entertainment app, and 1 categorized as other.

**Table 1:** Sexual health content of sex-related apps (N=137).

Variable		Primary purpose of apps					Sexual health content		
		Sex aid/ exploration	Entertainment	Sex education/ information	Sexual assault	Others	None	Contraception/ STI^a^ information	Sexual assault/ violence
**Total apps, n (%)**									
	iTunes	33 (35.8)	21 (22.8)	8 (8.6)	16 (17.3)	14 (15.2)	68 (73.9)	10 (10.8)	14 (15.2)
	Google Play	9 (20.0)	11 (24.4)	4 (8.8)	3 (6.6)	18 (40.0)	39 (86.6)	5 (11.1)	1 (2.2)
**Apps containing STI or contraception information, n (%)**									
	iTunes	2 (2.1)	0 (0)	7 (7.6)	0 (0)	1 (1.0)	N/A^b^	N/A	N/A
	Google Play	0 (0)	1 (2.2)	4 (8.8)	0 (0)	0 (0)	N/A	N/A	N/A
**App store category, n (%)**									
	Entertainment	14 (53.8)	11 (42.3)	0 (0)	0 (0)	1 (3.8)	26 (100)	0 (0)	0 (0)
	Games	3 (42.8)	3 (42.8)	0 (0)	1 (14.2)	0 (0)	6 (85.7)	0 (0)	1 (14.2)
	Education	0 (0)	3 (20.0)	3 (20.0)	5 (33.3)	4 (26.6)	10 (66.6)	4 (26.6)	6 (40.0)
	Books/ reference	1 (20.0)	1 (20.0)	1 (20.0)	2 (40.0)	0 (0)	3 (60.0)	1 (20.0)	1 (20.0)
	Health and fitness	7 (35.0)	0 (0)	4 (20.0)	1 (5.0)	8 (40.0)	16 (80.0)	4 (20.0)	1 (5.0)
	Lifestyle	13 (32.5)	11 (27.5)	2 (5.0)	4 (10.0)	10 (25.0)	34 (85.0)	5 (12.5)	2 (5.0)
	Medical	0 (0)	1 (10.0)	2 (20.0)	1 (10.0)	6 (60.0)	8 (80.0)	1 (10.0)	1 (10.0)
	Tools/ utilities	1 (20.0)	0 (0)	0 (0)	2 (40.0)	2 (40,0)	3 (60.0)	0 (0)	2 (40.0)
	Others	2 (22.2)	2 (22,2)	0 (0)	2 (22.2)	3 (33.3)	9 (100)	0 (0)	1 (11.1)
**User rating, n (%)**									
	Unrated	27 (31.7)	21 (24.7)	8 (9.4)	15 (17.6)	14 (16.4)	70 (82.3)	9 (10.5)	14 (16.4)
	1–3.9	12 (41.3)	7 (24.1)	2 (6.8)	2 (6.8)	6 (20.6)	25 (86.2)	3 (10.3)	1 (3.4)
	4–5	2 (8.6)	4 (17.3)	3 (13.0)	1 (4.3)	13 (56.5)	20 (86.9)	3 (13.0)	0 (0)

^a^STI: sexually transmissible infections.

^b^N/A: not applicable.

Of the 15 apps offering information about sexual assault, 5 (33%) had information regarding management after sexual assault, 5 (33%) had general information about sexual assault, 1 (7%) focused on identifying sexual assault victims, and the other 4 (27%) had information about sexual assault prevention. However, none of the apps with the primary purpose of sexual assault/violence contained any information about STIs or contraception.

We also recorded the number of downloads of apps from Google Play marketplace; this information is readily available within the marketplace and is displayed in a range (for example: between 1000 and 5000). We found that the 4 Android apps that contained sexual health information were downloaded less frequently than other sex aid or entertainment apps (the number of downloads is available in Multimedia Appendix 1).

### Part 2: Dating Apps

We included the first 50 free dating apps from each store, then excluded 25 duplicates from the list (Multimedia Appendix 2). In the analysis we excluded the apps that were not available for download (n=5), that required in-app purchases to proceed to basic functions such as receiving messages and online chats (n=7), and apps that did not function after 3 attempts (n=6).

**Figure 2:**
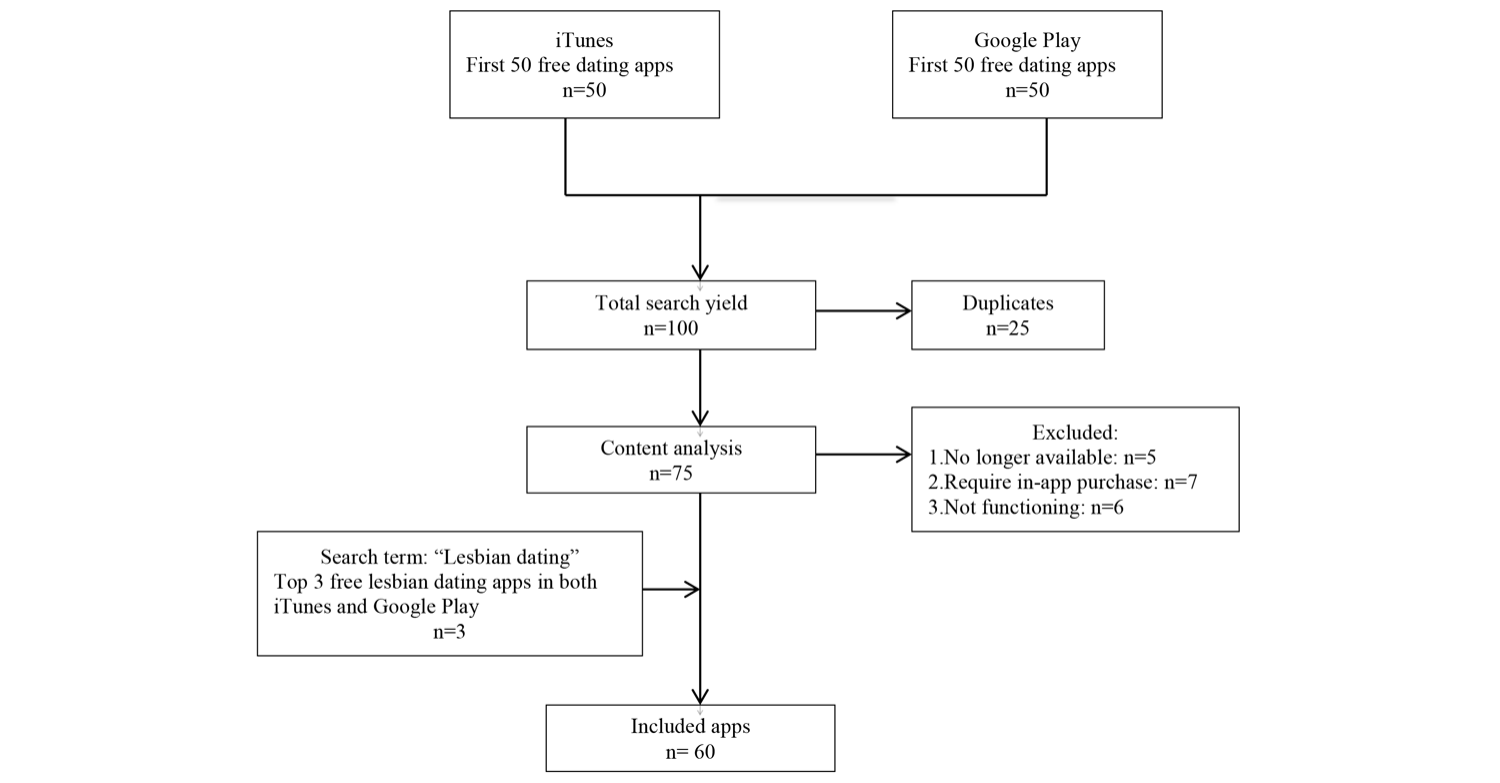
Inclusion of dating apps.

During initial review we realized that none of these apps targeted lesbian women, so we included the 3 top lesbian dating apps available in both the Apple iTunes store and the Google Play marketplace, found using the search term “lesbian dating.” We included 60 apps in the study (Figure 2). Of these, 44 (73%) apps targeted heterosexual users, 9 (15%) targeted MSM, 3 (5%) targeted lesbians, and 4 (7%) were for group dating and finding partners for threesomes (Table 2).

**Table 2:** Results of dating app review (N=60).

Variable		Target group				Sexual health content	Safe dating tips	Total
		Heterosexual	MSM^a^	Lesbian	Other^b^			
**Total apps, n (%)**								
	iTunes	28 (68)	9 (22)	3 (7)	1 (2)	7 (17)	6 (15)	41 (100)
	Google Play	16 (84)	0 (0)	0 (0)	3 (16)	2 (11)	1 (5)	19 (100)
**App store category, n (%)**								
	Social networking	34 (71)	9 (19)	3 (6)	2 (4)	8 (17)	5 (10)	48 (100)
	Communication	1 (100)	0 (0)	0 (0)	0 (0)	0 (0)	0 (0)	1 (100)
	Entertainment	1 (100)	0 (0)	0 (0)	0 (0)	1 (100)	0 (0)	1 (100)
	Lifestyle	8 (80)	0 (0)	0 (0)	2 (20)	0 (0)	1 (10)	10 (100)
**User star rating, n (%)**								
	N/A^c^	22 (65)	8 (24)	3 (9)	1 (3)	5 (15)	5 (15)	34 (100)
	1–3.9	8 (89)	0 (0)	0 (0)	1 (11)	1 (11)	0 (0)	9 (100)
	4–5	14 (82)	1 (6)	0 (0)	2 (12)	3 (18)	2 (12)	17 (100)

^a^MSM: men who have sex with men.

^b^Other includes threesome or group dating.

^c^N/A: not available.

We found that 9 (15%) of the 60 apps included sexual health content. The sexual health content was displayed in the apps in four different ways (Textbox 2). The majority of the apps with sexual health content targeted MSM (7/9, 78%) (Table 3). Only 1 heterosexual app contained sexual health content (Table 3). None of the 3 lesbian-specific apps contained any sexual health or safe dating content (Table 3). We found safe dating tips in 7 apps (12%), which included information such as “do not disclose your true identity online,” “always meet in public places,” and “trust one’s own instinct.” All the apps with safe dating tips targeted heterosexual users. The availability of sexual health content and safe dating tips was related to the target group of the apps (*P*<.001).

**Sexual health content within dating apps.**

- Preference of safe sex in users’ profiles (always, depends, or never)
- Pop-up messages encouraging sexually transmissible infection (STI) testing
- STI status in users’ profiles
- Links to or articles about STI information in apps or websites

**Table 3:** Availability of sexual health content and safe dating tips in dating and entertainment apps according to target group.

Target group	Content, n (%)			*P* value	Total no. of apps
	Sexual health content	Safe dating tips	None		
Heterosexual	1 (2)	7 (16)	36 (81)	<.001	44
MSM^a^	7 (78)	0 (0)	2 (22)		9
Lesbian	0 (0)	0 (0)	3 (100)		3
Other	1 (25)	0 (0)	3 (75)		4

^a^MSM: men who have sex with men.

## Discussion

### Principal Findings

Our review identified a large number of smartphone apps that have sexual content or promote dating. We found that the majority of these sex-related and dating apps contained no information about sexual health promotion. We chose to review all apps found using our search terms, regardless of app store category, rather than focusing on apps that aim to provide sexual health information and education. The reason behind this decision was that previous research has shown that apps designed for educational purposes are infrequently downloaded and underused [23]. We made the same observation in our review. For example, *Sexually Transmitted STDs*, an app that provides information about STIs, including definition, transmission, symptoms, treatments, and prevention, had fewer than 1000 downloads to January 2015. On the other hand, some game apps were downloaded far more frequently; for example, *Bliss –* *The Game for Lovers* was downloaded more than 100,000 times from the Android Google Play marketplace (this app is also available in iTunes). This finding demonstrates the advantage of integrating sexual health information within popular entertainment apps in order to reach out to more people. We found that 73% of the apps with sexual health content had the primary purpose of education, which means that they might not be attractive to people who are not specifically seeking out sexual health information. Only 2 sex aid apps and 1 entertainment app contained sexual health content, demonstrating room for improvement. We hypothesize that exposing users to sexual health content while they are using these sex-related apps (presumably while they are thinking about sex or during foreplay) might be a good way to remind them of safe sex practices. This hypothesis will need to be examined in future research.

In the first part of the study, we found that 19 sexual apps had the primary purpose of providing support and information for sexual assault or violence. These apps offered a variety of information about topics including mental health support after sexual assault, prevention of sexual assault, and general information regarding sexual assault. However, none mentioned the potential adverse sexual health outcomes faced by sexual assault victims. The risk of unwanted pregnancy and contracting STIs, and steps that can be taken to prevent or treat these, are critical in the aftercare of sexual assaults [25]. Many victims of sexual assault do not seek help from health professionals [25]; therefore, providing such information via smartphone apps might encourage victims to seek medical advice for STI and pregnancy prevention.

Our review of dating apps showed that very few included any sexual health content (9 of 60 dating apps reviewed, 15%). The majority of these apps were targeted at an MSM population (7 out of 9). These findings suggest that there is more focus on the sexual risks of MSM who use dating apps than other groups. MSM are disproportionately affected by HIV globally and are a key population for HIV infection and STI prevention [26-28]. These geosocial-networking apps can potentially function as an entry point for HIV intervention delivery, assisting health professionals to reach the key populations, particularly where populations are hidden or difficult to access [7,15]. Delivering sexual health interventions via dating apps is an important area to be addressed in prevention of HIV infection.

It is unfortunate that the opportunity to deliver messages to other groups via these apps is being missed. Only 1 of the 44 heterosexual dating apps reviewed had any sexual health content (STI status in users’ profile). People who use dating apps seem likely to have more casual sexual partners than people who do not, which means higher risk of contracting STIs [13]. While sexual health content appears to be acceptable to MSM who use dating apps, its acceptability among other groups is unknown [15,24]. More work needs to be done to increase the sexual risk awareness of users of heterosexual dating apps.

Dating apps used four different modes to display sexual health content: pop-up messages, infection status in users’ profiles, safe sex preferences in users’ profiles, and blog posts or links to sexual health information in the apps or linked websites. Each of these messages has its own limitations in reminding users of their sexual risk. First, the frequency and timing of pop-up messages might influence users’ acceptance. If the frequency is too high, it might desensitize users. Messages appearing during chats could cause annoyance, which could lead to users unsubscribing and turning to other apps that exclude these kinds of messages. Second, having HIV or STI status and safe sex preference on a profile can be a good way to assist users’ partner filtering processes. Nevertheless, these messages are highly dependent on users’ self-reports and their knowledge of infection status. These disclosures may also expose users to stigma and discrimination or cyberbullying [29]. In the 2 apps that enabled indication of preference for safe sex practice, the concept was not defined. Moreover, since this information appears on users’ profiles, users can decide to disclose the status or preference, or not. Third, in-app blog posts can be a good place to display information regarding STIs and HIV if these posts are updated frequently and the information provided is correct. On the other hand, having links to sexual health clinics in the websites rather than within the apps might be less effective, since users have to be actively looking for sexual health information and using the website at the same time to be exposed to these messages. This type of message is less likely to effectively remind users about their sexual risks. Further research is needed to understand the impact these messages have on users’ behavior and health outcomes.

We identified another potential platform for intervention during the app review: the advertisement space within apps. Advertisements (ads) mostly exist in two forms: pop-up ads and ads that appear on the bottom of the screen; users can close pop-up ads, but they usually cannot remove bottom-of-the-screen ads. Once users click on the ads, they will usually be directed to a new page that contains more information about the product being advertised (most likely another paid app). Health promoters could purchase these ad spaces to display sexual health information or links. Some app developers sell ad spaces as pop-ups for advertisers to purchase. For example, Grindr sells mobile Web banner ads, which can link directly to advertisers’ websites, emails, or mobile numbers [30]. These ads are sold as cost per thousand banner impressions, with the price ranging from USD $9 to $25 per thousand banner impressions for iPhone and Android devices. It is potentially a cost-effective way of promoting sexual health, as it is cheaper and more focused on target groups than traditional media ads. However, the limitation of this method is that users are usually encouraged to subscribe to premium membership (by paying a monthly fee or upgrading to the paid version of the apps) in order to avoid seeing the ads. Once users upgrade to the paid versions, they might no longer be exposed to sexual health information through this medium. More research is needed to evaluate the effectiveness of advertising through in-app ads and how to make sure all users receive the messages being advertised.

Research has shown that 80% of Internet users in the United States search online for health information, and that young people are gathering health information using mobile devices with increasing frequency, including sexual health information [31]. However, while new technologies, including smartphone apps, are used to facilitate health information seeking, health-related apps are infrequently downloaded and rarely used [32]. This suggests that, to promote sexual health through smartphone apps, researchers could partner with app developers in order to integrate sexual health promotion interventions in popular sex-related or dating apps [24]. Such partnerships will be difficult to form when the interests of the parties conflict. For dating app developers, sexual health content that reminds users of their sexual risk might be unattractive, as it could jeopardize their popularity among users [24]. However, it is evident that these apps provide novel opportunities to engage at-risk populations in sexual health interventions [7,8,11,15,24].

Several studies have suggested that young adults consider sexual health promotion via apps acceptable [10,15,24]. Sun et al found that approximately two-thirds of MSM were willing to receive sexual health-related information through apps, and 26% of them requested referrals for HIV and STI testing [15]. The willingness to participate in future HIV infection and STI prevention programs is even higher among MSM aged between 19 and 24 years [24]. Holloway et al found that 80% of young MSM recruited through Grindr expressed an interest in joining such programs, and 71% preferred to have the information delivered through smartphone apps [24].

### Limitations and Strengths

Our study had some limitations. First, smartphone apps are changing rapidly, including their content, popularity, and even availability. The ranking of popularity varies over time; therefore, our search results might be different if repeated. Updates of the apps can change apps’ features and functions, including the sexual health content that we looked for. For example, since our review, Tinder has agreed to provide information for STI testing locations [33]. Second, our categorization of pop-up sexual health content might have been inaccurate: we could have missed infrequent pop-up messages, or those appearing only around major events. We used only 1 device for each platform, which prevented us noticing variation in app function between devices (if any). We also did not identify any differences in the frequency or availability of sexual health content using different profiles. Third, the terms “sexual” and “dating” used to search app stores for sex-related apps and dating apps may have constrained our search. Other terms such as “sex” or “networking” could be considered for future searches. Fourth, our search was limited to the Apple iTunes store and the Android Google Play store, and thus neglected apps from other smartphone operating systems (eg, Microsoft, Palm, Blackberry). However, this decision was justified by the fact that 96% of smartphone users worldwide use either Apple or Google operating systems [34].

Despite these limitations, this study is, to our knowledge, the first to review the inclusion of sexual health content within sexual and dating apps that are not primarily aimed at sex education. We are unsure how much influence these messages have on users. Further research in this field is needed to understand the effectiveness and efficiency of promoting sexual health through in-app messages.

### Conclusions

The majority of sex-related and dating smartphone apps do not contain any sexual health content, with the exception of dating apps targeting MSM. Using smartphone apps to promote sexual health is a potentially important method of reaching at-risk populations. Due to the low rate of integration of sexual health information in dating apps and sex-related entertainment apps, we suggest that sexual health researchers work with app developers to promote sexual health within existing popular apps. Further investigation of the acceptability and effectiveness of sexual health content in sexual and dating apps is needed.

## Appendix

Multimedia Appendix 1 List of sexual apps.

Multimedia Appendix 2 List of dating apps.

## Abbreviations

HIV: human immunodeficiency virus
MSM: men who have sex with men
STI: sexually transmissible infections
